# Discordance between chromatin accessibility and transcriptional activity during the human primed-to-naïve pluripotency transition process

**DOI:** 10.1186/s13619-023-00179-2

**Published:** 2023-11-08

**Authors:** Zhifen Tu, Yan Bi, Tengyan Mao, Hong Wang, Shaorong Gao, Yixuan Wang

**Affiliations:** 1 Translational Medical Center for Stem Cell Therapy & Institute for Regenerative Medicine, Shanghai East Hospital, School of Life Sciences and Technology, Tongji University, Shanghai, 200120 China; 2 Frontier Science Center for Stem Cell Research, Tongji University, 1239 Siping Road, Shanghai, 200092 China; 3 Shanghai Key Laboratory of Maternal Fetal Medicine, Clinical and Translational Research Center of Shanghai First Maternity and Infant Hospital, School of Life Sciences and Technology, Tongji University, Shanghai, 200092 China

**Keywords:** Naïve pluripotency, Primed-to-naïve transition, Dual reporter system, Discordance between chromatin accessibility and transcriptional activity, Histone modifications

## Abstract

Naïve pluripotent state can be obtained by several strategies from various types of cells, in which the cell fate roadmap as well as key biological events involved in the journey have been described in detail. Here, we carefully explored the chromatin accessibility dynamics during the primed-to-naïve transition by adopting a dual fluorescent reporter system and the assay for transposase-accessible chromatin (ATAC)-seq. Our results revealed critical chromatin remodeling events and highlight the discordance between chromatin accessibility and transcriptional activity. We further demonstrate that the differential epigenetic modifications and transcription factor (TF) activities may play a critical role in regulating gene expression, and account for the observed variations in gene expression despite similar chromatin landscapes.

**Supplementary Information:**

The online version contains supplementary material available at 10.1186/s13619-023-00179-2.

## Background

Human naïve pluripotent stem cells (PSCs) represent the ground state of pluripotency, corresponding to the preimplantation epiblast (Hackett and Surani 2014; Huang et al. 2014; Nichols and Smith 2009; Pera 2014). These cells display greater plasticity and an unbiased potential for differentiation compared to conventional PSCs in the primed pluripotent state (Guo et al. 2021; Lee et al. 2017; Yang et al. 2016), thus offering a valuable tool for developmental studies and potential therapeutic applications. The acquisition of naïve pluripotency can be achieved through the derivation of preimplantation embryos, reprogramming of somatic cells, or transitioning of conventional PSCs in the primed state (Bayerl et al. 2021; Chan et al. 2013; Chen et al. 2015; Gafni et al. 2013; Giulitti et al. 2019; Guo et al. 2016; Liu et al. 2017; Pastor et al. 2016; Qin et al. 2016; Szczerbinska et al. 2019; Takashima et al. 2014; Theunissen et al. 2014; Ware et al. 2014). In our previous study, we detailed a high-resolution cell roadmap towards human naïve pluripotency from primed PSCs with the appearance of trophectoderm (TE) and primitive endoderm (PrE) signatures (Bi et al. 2022). We also elucidated the cell fate transitions from the primed to naïve pluripotency by our dual fluorescent reporter system via integration of transcription profiles and the chromatin accessibility landscape. However, further in-depth investigation is still needed to fully understand the dynamics of chromatin landscape during the primed-to-naïve transition.

The advancement in high-throughput sequencing technologies has led to the development of various assays aimed at deciphering the epigenetic landscape, including Assay of Transposase Accessible Chromatin sequencing (ATAC-seq) (Buenrostro et al. 2013, 2015), DNase I hypersensitive sites sequencing (DNase-seq) (Boyle et al. 2008; Song and Crawford 2010; Thurman et al. 2012) and Formaldehyde-Assisted Isolation of Regulatory Elements sequencing (FAIRE-seq) (Giresi et al. 2007). Among these assays, ATAC-seq demonstrates superior sensitivity and specificity compared to FAIRE-seq, while exhibiting comparable sensitivity and specificity to DNase-seq (Buenrostro et al. 2013). Additionally, ATAC-seq requires lower input material, has a shorter assay time and can even be applied to frozen tissue samples (Corces et al. 2017). Moreover, the advanced downstream analyses including chromatin accessibility, enhancer landscapes, motif enrichment, nucleosome positioning, TF footprints and further integration with multi-omics data to reconstruct regulatory networks, allow for robust sequencing analyses and accurate biological results. Thus, ATAC-seq is widely used for obtaining chromatin landscape information among assays designed for measuring chromatin accessibility currently, with an increasing number of curated ATAC-seq datasets and publications.

Chromatin can switch dynamically between transcriptionally active euchromatin and inactive heterochromatin in biological events. Chromatin remodeling is closely related to gene expression regulation. Multiple studies have established a high correlation between chromatin accessibility and gene expression, with increased chromatin opening being correlated with higher gene expression levels, and vice versa (Cao et al. 2018; Li et al. 2017; Pastor et al. 2018; Wu et al. 2016, 2018; Yu et al. 2020). For example, the promoter accessibility measured by ATAC-seq in early human embryo development exhibited high correlation with gene activities, including the pluripotency related genes *POU5F1* and *NANOG*, as well as the EGA genes *ZSCAN5B*, etc. (Wu et al. 2018); the activation of genes at various stages of mouse embryo development was highly correlated with increased levels of promoter ATAC-seq signals (Wu et al. 2016); Similar results could also be found in the mouse BiPNT (BMP4 induced primed-to-naïve transition) and TFiPNT (TFs induced primed-to-naïve transition) process, showing concordant gene expression and ATAC signals (Yu et al. 2020). However, recent evidence has also described the discordance between chromatin accessibility and transcriptional activity and concluded that the regulation of transcription is involved with many underlying mechanisms (Kiani et al. 2022).

In this study, we observe that the chromatin remodeling events, including the opening of naïve specific chromatin enriched with motifs for the OCT/SOX/KLF families, already occurred in RFP-negative cells despite the absence of transcriptional activity related to naïve pluripotency. Further analysis of the discordance between transcription profiles and chromatin accessibility indicated the importance of epigenetic modifications and TF activities in gene expression regulation. Overall, our study provides new insights into the dynamics of chromatin landscape and the global regulation of transcriptional activity.

## Results

### The dynamics of chromatin accessibility during the primed-to-naïve transition

**Fig. 1:**
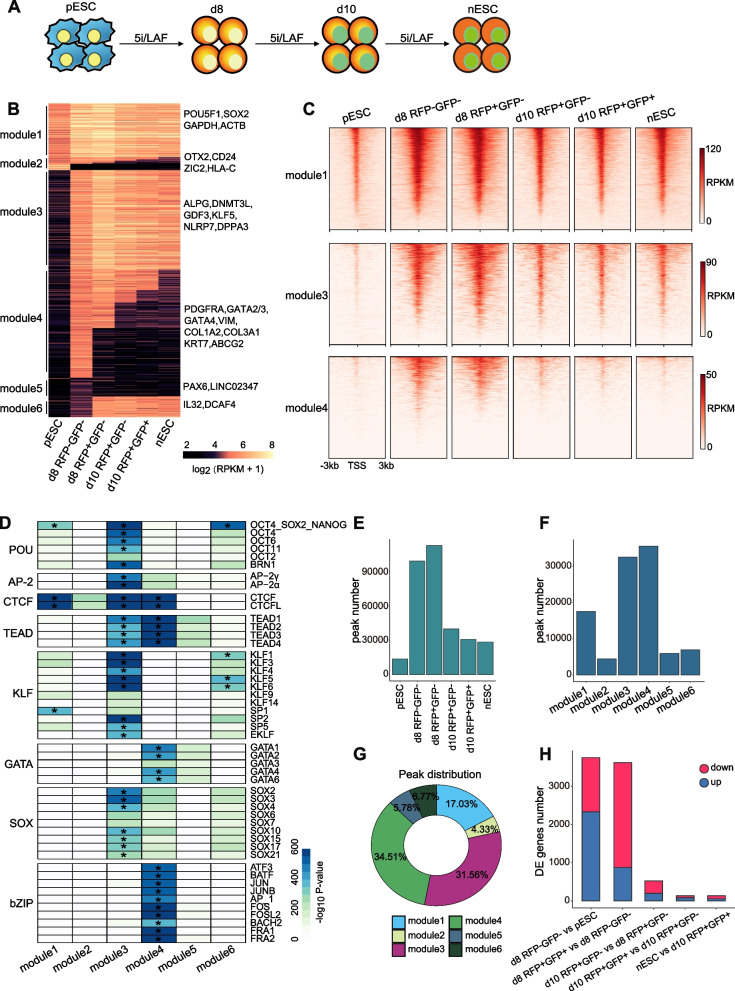
Chromatin accessibility dynamics during the primed-to-naive transition. **A** Schematic representation of the primed-to-naive transition using 5iLAF culture conditions. **B** Chromatin loci arranged into groups according to closed or open status during the putative consecutive stages towards naive pluripotency. Representative genes are noted for each module on the right side. **C** Heatmaps of ATAC-seq signals for module 1, 3, 4, respectively. The heatmaps are centered on the ATAC-seq peak (upstream 3 kb and downstream 3 kb of the peaks). **D** Motif discovery for each module shown in Fig. 1 B. *p < 10^^ −300^. **E** The number of peaks defined in each sample. **F** The number of peaks defined in each module. **G** Pie chart of the peak distribution for each module. **H** The number of differentially expressed genes between putative consecutive stages

In our previous study, we investigated the establishment of human naïve pluripotency from cells in the primed state upon 5iLAF culture. We utilized a dual fluorescent reporter system consisting of *ALPG*-promoter-RFP and *OCT4*-ΔPE-GFP, to monitor the fluorescence dynamics throughout this process. The *OCT4*-ΔPE-GFP (ΔPE: delta proximal enhancer) reporter system was specifically designed to indicate naïve pluripotency via the *OCT4* distal enhancer employment and to establish the 5iLAF culture condition. Presently, this method remains a prevalent approach for monitoring naïve pluripotency (Theunissen et al. 2014). The *ALPG*-promoter-RFP reporter was developed in our previous study to trace the *ALPG* expression, and we found that it could effectively indicate the establishment of naïve pluripotency during naïve reprogramming or naïve-to-primed conversion process (Bi et al. 2020). Moreover, we demonstrated that the dual reporter system could monitor naïve pluripotency comprehensively (Bi et al. 2020, 2022). Analysis using flow cytometry revealed the emergence of the *ALPG* (RFP^+^) subpopulation on day 8 and the appearance of the GFP^+^ subpopulation on day 10. Transcriptional analysis of the transitioning intermediates representing distinct fluorescence signals from our earlier studies revealed that the RFP-positive cells shared great similarity with naïve ESC (nESC), while RFP^+^GFP^+^ cells nearly mirror nESCs in terms of their transcriptome (Bi et al. 2022). The proportion of RFP^+^GFP^+^ cells increased along with the induction process, and naïve-like colonies were picked to establish nESC lines (Fig. 1A).

To illustrate the chromatin accessibility landscape during the primed-to-naïve transition process, we performed ATAC-seq on the transitioning intermediates collected based on their fluorescence dynamics. To gain a deeper understanding of the chromatin accessibility dynamics (CAD) during the primed-to-naïve transition process, we first established the global threshold and defined the cut-off between open and closed chromatin by controlling the FDR (false discovery rate) value based on the background regions (Fig. S1A, Methods section). Subsequently, the chromatin accessibility dynamics were summarized according to chromatin state. The CAD charting and heatmaps revealed that the chromatin dynamics can be classified into six modules, representing distinct chromatin landscape (Fig. 1B-C, Fig. S1B, Table S1). Additionally, the expression dynamics of representative genes from each module were displayed (Fig. S1C). Together with the GO analysis for genes within each module (Fig. S1D), we discovered that the loci of genes involved in DNA repair and chromatin organization remained permanently open (module 1), including those of the shared pluripotency-related genes *POU5F1*, *SOX2* and housekeeping genes *GAPDH* and *ACTB*. The loci of genes within module 2, associated with neural system development and regulation of growth, underwent an open-to-closed transition, including the primed state-specific factor *OTX2* and *ZIC2*. We also observed that the loci of naïve pluripotency-related genes, involved in embryonic morphogenesis and blastocyst development, such as *ALPG*, *DNMT3L*, *NLRP7* and *DPPA3*, were opened since day 8 (module 3), indicating that the naïve specific chromatin landscape may have been established in RFP-negative cells on day 8 during the primed-to-naïve transition process. Furthermore, the loci of genes including both TE markers (*GATA3*, *KRT7*, etc.) and PrE markers (*GATA4*, *PDGFRA*, etc.), specified in module 4, were opened in RFP-negative cells on day 8 and then underwent an open-to-closed transition, suggesting the appearance of TE signatures and PrE signatures. GO analysis of the genes within module 4 also showed that these genes are involved in cell morphogenesis and response to growth factor. Additionally, the loci of differentiation-associated genes, such as *PAX6*, remained permanently closed (module 5). The loci within module 6 were mainly distributed in distal intergenic and intron areas, associated with actin cytoskeleton organization and gamete generation. The peak annotations of each module were also displayed to provide a comprehensive overview for peak distribution (Fig. S1E).

Next, we conducted a motif enrichment analysis to identify the potential regulatory network of transcription factors for each module (Fig. 1D). Results showed that motifs for *CTCF* and *OCT4-SOX2-NANOG* were significantly enriched in module 1. The loci within module 3 exhibited a high level of enrichment with motifs for TFs from the AP-2, CTCF, POU, KLF, SOX and TEAD families, indicating the remodeling of naïve specific chromatin. Motifs for CTCF and TEAD families were significantly enriched in module 4. Specifically, we observed that the motifs for *GATA1/2/3/4/6* and bZIP family members, including *JUN*, *FOSL2* and *FOS*, were specifically enriched in module 4, consistent with our previous findings that indicated the appearance of TE and PrE signatures during the primed-to-naïve transition process. Together, these results suggest that the loci involved in the chromatin remodeling events during the primed-to-naïve transition process, such as the opening of naïve specific chromatin and the temporary activation of TE and PrE signatures, were already present in the RFP-negative cells, and that the RFP-positive cells shared a similar chromatin landscape with RFP-negative cells on day 8 (Fig. 1B and D).

### Discordance between chromatin accessibility and transcriptome

**Fig. 2:**
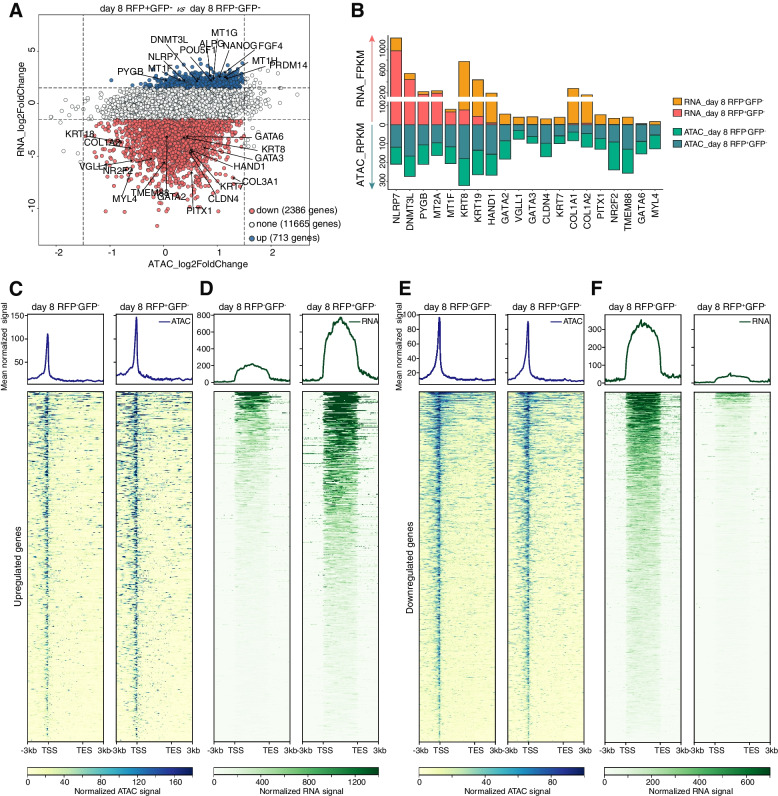
Profiles discordance between chromatin accessibility and transcriptional activity. **A** Scatter plot of the comparison between RNA signals and ATAC signals for the loci of genes within chromatin that was open in both RFP-negative cells and RFP-positive cells on day 8. **B** Bar plot of the corresponding RNA signals and ATAC signals for representative genes. **C** Heatmaps and pileups of ATAC signals for the discordant upregulated genes (upstream 3 kb and downstream 3 kb of the peaks). **D** Heatmaps and pileups of RNA signals for the discordant upregulated genes (upstream 3 kb and downstream 3 kb of the transcripts with merged exons). **E** Heatmaps and pileups of ATAC signals for the discordant downregulated genes (upstream 3 kb and downstream 3 kb of the peaks). **F** Heatmaps and pileups of RNA signals for the discordant downregulated genes (upstream 3 kb and downstream 3 kb of the transcripts with merged exons)

Our CAD charting revealed marked differences in the chromatin landscape between pESCs and RFP-negative cells on day 8, with closing of the loci associated with primed pluripotency and opening of specific chromatin associated with naïve pluripotency, TE and PrE signatures (Fig. 1B and D). The peak number statistics showed that chromatin was significantly accessible in both RFP-negative and RFP-positive cells on day 8, and then some temporarily accessible chromatin became closed as the induction process progressed and eventually, the cells reached the naïve state (Fig. 1E). According to the statistics of peaks in each module, we also found that a considerable proportion of peaks was in module 1 (17.03%), module 3 (31.56%) and module 4 (34.51%) (Fig. 1F-G). Together with the large peaks overlap and similar peak signal strength (Fig. 1B, Fig. S1F-G), we concluded that the RFP-positive cells shared the similar chromatin landscape with RFP-negative cells on day 8. We further investigated the dynamics of gene expression during the primed-to-naïve transition process. Surprisingly, we observed a discrepancy between chromatin accessibility and transcriptional activity. The numbers of differentially expressed genes between consecutive stages revealed substantial differences in transcriptome between RFP-negative cells and RFP-positive cells on day 8 despite the similar chromatin landscape (Fig. 1H), indicating that many genes may share similar chromatin state but differential expression levels. To further investigate this phenomenon, we then focused on comparing the signals of peaks opened in both RFP-negative cells and RFP-positive cells on day 8 and the corresponding genes expression between these two cells. (Fig. 2A-B).

We observed two categories of genes exhibit a discordant profile between chromatin accessibility and gene expression. One group of genes enriched with naïve markers displayed higher expression levels in RFP-positive cells compared to those in RFP-negative cells, such as *NLRP7*, *DNMT3L*, and *PYGB*, while the other group of genes enriched with TE and PrE markers exhibited higher expression in RFP-negative cells than RFP-positive cells, such as *GATA2/3*, *PITX1* and *GATA6*. Both groups showed similar normalized ATAC-seq signals. Furthermore, we presented a detailed analysis of chromatin accessibility and gene expression levels for both categories of genes (Fig. 2C-F, Fig. S2).

Taken together, these results provide further evidence for the profile discordance between chromatin accessibility and gene expression, suggesting that transcriptional activity may be regulated at multi-omics levels, beyond the corresponding chromatin accessibility.

### Histone modifications play an important role in regulation of transcriptional activity

Histone modifications play a crucial role in regulating gene expression through the establishment of global chromatin environments and the orchestration of chromatin structure (Hyun et al. 2017; Kouzarides 2007; Liu et al. 2016). Considering the association of H3K9me3/H3K27me3 with heterochromatin and the link between H3K4me3/H3K27ac and active/opened chromatin state, we subsequently wondered whether the active histone modifications H3K4me3 and H3K27ac, associated with gene activation, could interpret the observed discordance between chromatin accessibility and transcriptional activity in this study. To this end, we mapped the genome-wide profiles of H3K4me3 and H3K27ac in RFP-negative cells and RFP-positive cells on day 8 by using an ultra-low-input micrococcal nuclease-based native chromatin immunoprecipitation (ULI-NChIP) method (Table S2) (Brind’Amour et al. 2015).

**Fig. 3:**
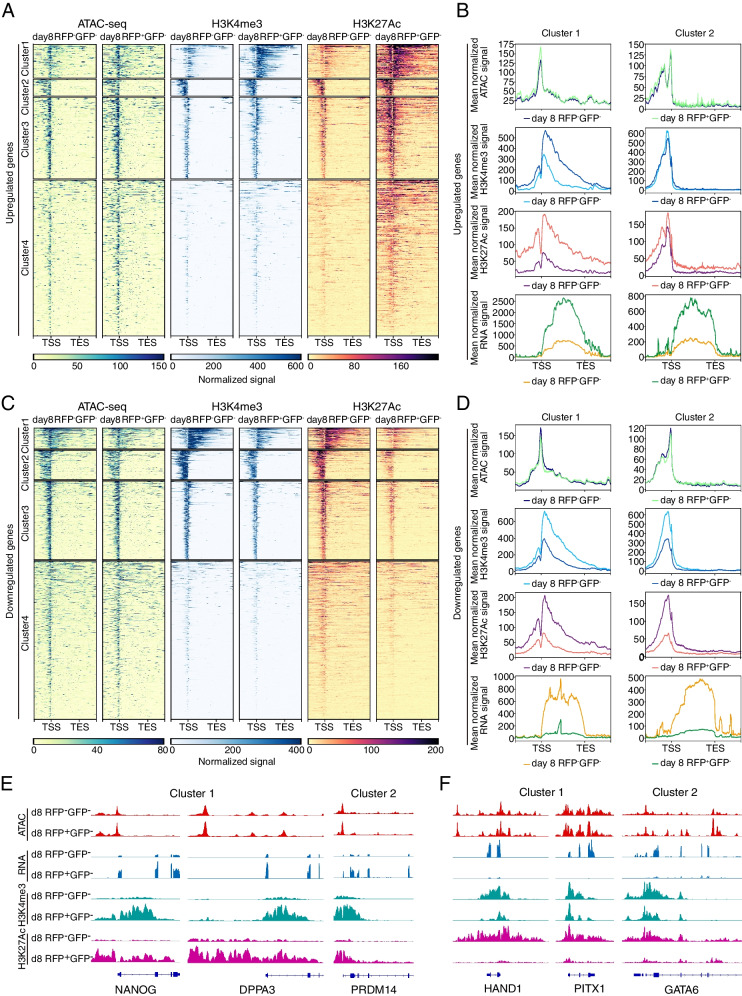
Histone modifications profiles of the discordant genes. **A** Heatmaps of ATAC signals (left), H3K4me3 signals (middle) and H3K27ac signals (right) for the discordant upregulated genes (upstream 3 kb and downstream 3 kb of the peaks). Loci were clustered by the k-means algorithm based on the signal profile and arranged by the average signal strength. **B** Pileups of mean ATAC signals (first panel), mean H3K4me3 signals (second panel), mean H3K27ac signals (third panel) and mean RNA signals (fourth panel) for the genes within cluster 1 and cluster 2 showed in Fig. 3 A (upstream 3 kb and downstream 3 kb of the peaks or transcripts with merged exons). **C** Heatmaps of ATAC signals (left), H3K4me3 signals (middle) and H3K27ac signals (right) for the discordant downregulated genes (upstream 3 kb and downstream 3 kb of the peaks). Loci were clustered by the k-means algorithm based on the signal profile and arranged by the average signal strength. **D** Pileups of mean ATAC signals (first panel), mean H3K4me3 signals (second panel), mean H3K27ac signals (third panel) and mean RNA signals (fourth panel) for the genes within cluster 1 and cluster 2 showed in Fig. 3C (upstream 3 kb and downstream 3 kb of the peaks or transcripts with merged exons). **E** ATAC signals (first panel), RNA signals (second panel), H3K4me3 signals (third panel) and H3K27ac signals (fourth panel) for the representative loci within cluster 1 and cluster 2 showed in Fig. 3A. **F** ATAC signals (first panel), RNA signals (second panel), H3K4me3 signals (third panel) and H3K27ac signals (fourth panel) for the representative loci within cluster 1 and cluster 2 showed in Fig. 3C

Integrative analysis of chromatin accessibility, histone modifications and transcriptome showed that H3K4me3 and H3K27ac modifications have a significant impact on gene expression (Fig. 3A-D, Fig. S3). In the group of genes with higher expression levels in RFP-positive cells compared to RFP-negative cells, stronger signals of the H3K4me3 and H3K27ac modifications were observed, especially for the genes within cluster 1, 2 and 3 (Fig. 3A-B). The reverse trend was also observed in the group of genes showing lower expression levels in RFP-positive cells compared to RFP-negative cells as well as weaker signals of H3K4me3 and H3K27ac (Fig. 3C-D). Specifically, the signals of ATAC-seq, RNA-seq and histone modifications were displayed for representative genes within each cluster of each group (Fig. 3E-F, Fig. S4).

Additionally, we observed a greater signal difference for H3K27ac compared to H3K4me3, suggesting that H3K27ac modification may play a more significant role in regulating gene expression in this scenario.

Altogether, our results suggest that despite similar chromatin landscapes, differences in epigenetic modifications may also exist, which may play an important role in determining the transcriptional activities. The dynamics of H3K4me3 and H3K27ac modifications on the corresponding chromatin are likely to influence the expression of genes.

### Differential TF activities may contribute to the observed discordance between chromatin accessibility and transcriptional activity

**Fig. 4:**
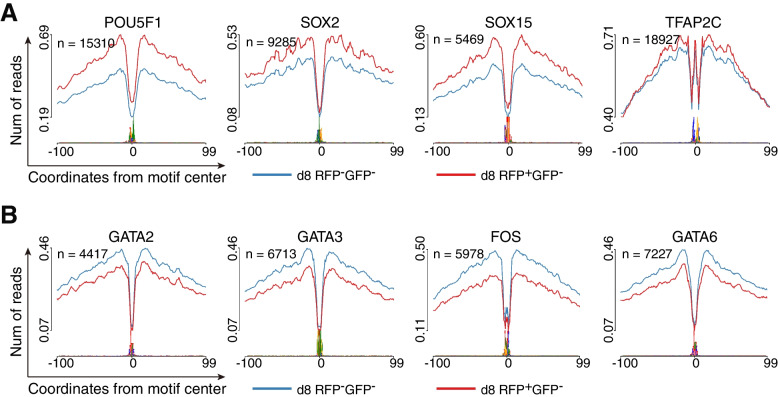
Transcription factors activities in both RFP-negative cells and RFP-positive cells on day 8. **A** Footprint profiles of transcription factors (TFs) generated by motif matching and differential analysis for POU5F1 (POU5F1-SOX2-TCF-NANOG motif), *SOX2* , *SOX15* and *TFAP2C*. **B** Footprint profiles of transcription factors (TFs) generated by motif matching and differential analysis for *GATA2*, *GATA3*, *FOS* and *GATA6*

To investigate the potential relationship between the discordance and the differential occupancy capacities of key transcription factors (TFs) on the accessible chromatin, we utilized the HINT-ATAC toolbox (Li et al. 2019) to perform footprint analyses of TF motifs in both RFP-negative and RFP-positive cells on day 8. We observed that *POU5F1* (with a POU5F1-SOX2-TCF-NANOG motif) displayed higher occupancy capacity and TF activity in RFP-positive cells compared to RFP-negative cells. A similar tendency was also observed for other TFs associated with pluripotency, such as *SOX2* and *SOX15* (Fig. 4A). On the other hand, the TE marker gene *GATA3*, *FOS* and PrE marker genes *GATA6* showed higher TF activity and occupancy capacity in RFP-negative cells than in RFP-positive cells (Fig. 4B). Notably, *TFAP2C*, the marker for both naïve pluripotency and TE, maintained high occupancy capacity in both RFP-positive and RFP-negative cells (Fig. 4A).

Taken together, the discordance between chromatin state and transcription activity observed in day 8 RFP-positive and RFP-negative cells may be related to the distinct activity levels and differential occupancy capacities of key TFs.

## Discussion

In this study, we depicted the chromatin accessibility landscape during the primed-to-naïve transition process by ATAC-seq. We found that the loci of naïve specific genes became accessible in RFP-negative cells since day 8, as well as the closing of primed specific chromatin and temporary opening of TE/PrE-related loci, consistent with our findings in the previous study (Bi et al. 2022). These results suggest that the majority of chromatin remodeling events during the primed-to-naïve transition process were accomplished in RFP-negative cells on day 8, which is also the time point for appearance of RFP-positive cells with detectable naïve signals. However, further investigation of gene expression dynamics in transcriptome revealed significant differences between RFP-negative cells and RFP-positive cells on day 8, including the lower expression of critical naïve pluripotency-related genes and higher expression of TE/PrE signature-related genes in RFP-negative cells compared to those in RFP-positive cells, despite the similar normalized ATAC signals and opened chromatin state. Actually, the naïve specific chromatin landscape may have been already established in RFP-negative cells on day 8, prior to the gene expression changes. This represents a chromatin priming phenomenon thought to be utilized by genes to ensure they are expressed correctly at time and levels (Bonifer and Cockerill 2017). These findings highlight the divergent transcriptional programs between the RFP-negative cells and RFP-positive cells, as well as the profiling discordance between chromatin accessibility and transcriptional activity.

Histone modifications are key epigenetic regulators that play important roles in various cellular processes, including gene expression, DNA replication and repair, and chromatin compaction (Kiani et al. 2022; Kouzarides 2007; Liu et al. 2016). These modifications can also alter the higher-order chromatin structure by affecting the contact between different histones in adjacent nucleosomes or the interaction of histones with DNA and recruitment of nonhistone proteins to further modify chromatin, such as remodeling ATPases. Of all the known modifications, acetylation has been considered to have the greatest capacity to disassemble chromatin, as it neutralizes the basic charge of lysine (Kouzarides 2007). Typically, H3K4me3 and H3K27ac modifications are considered as markers of active chromatin, reflecting active transcriptional activity.

To determine whether histone modifications contribute to the discrepancy between chromatin accessibility and transcriptional activity, we conducted the genome-wide profiles of H3K4me3 and H3K27ac modifications. Our results showed that genes with similar chromatin accessibility but distinct transcriptional activity also exhibit differential H3K4me3 and H3K27ac signals accordingly, suggesting the critical roles of histone modifications in gene expression regulation. Interestingly, H3K27ac modifications exhibited more pronounced differential signals, suggesting distinct chromatin structures despite similar chromatin landscape. Altogether, these results suggest that gene expression is regulated by both chromatin accessibility and epigenetic modifications on its corresponding chromatin.

A footprint in ATAC-seq is characterized by the binding pattern of an active TF to DNA, which impedes Tn5 cleavage at the binding site. This results in a relative decrease within the open chromatin region (Li et al. 2019; Yan et al. 2020). Thus, footprints of actively bound TFs are related to gene expression regulation and can be used to reconstruct a regulatory network. In our study, we evaluated the occupancy capacity of TFs on the open chromatin and found that PrE- and TE-related TFs displayed much higher levels of activity and occupancy capacities in RFP-negative cells. Conversely, naïve pluripotency-related TFs displayed robust activity in RFP-positive cells, suggesting a potential correlation between TF activity and transcriptional activity. Additionally, we also found that the knockdown of *NANOG* (with a POU5F1-SOX2-TCF-NANOG motif) could dramatically reduce the proportion of naïve populations during the primed-to-naïve transition process in our previous study (Bi et al. 2022).

In conclusion, we demonstrate a profiling discordance between chromatin accessibility and transcriptional activity, and illustrate that the epigenetic modifications and TF activities play an important role in regulating gene expression in addition to the chromatin accessibility. Our study may offer new insights for further exploration into the regulation of transcriptional activity.

## Methods

### Cell lines

The human primed ESCs (pESCs) with H9 background were kindly provided by Haoyi Wang, Institute of Zoology, CAS. pESCs genetically engineered with *ALPG*-promoter-RFP (RFP) and *OCT4*-△PE-GFP (GFP) were generated following previous research (Bi et al. 2022). All research with human cell lines in this study complied with the principles laid out in the International Society for Stem Cell Research and with ethical approval for these experiments by the Biological Research Ethics Committee of Tongji University.

### Cell cultures

The human cell lines were cultured at 37 °C, 5% CO_2_. Mycoplasma tests were performed every week. Human pESCs were maintained in conventional human ESC medium containing DMEM/F12 (Thermo Fisher) with 20% KnockOut SR (Thermo Fisher), 1% nonessential amino acids (Millipore), 2 mM GlutaMAX (Millipore), penicillin-streptomycin (Millipore), and 8 ng/ml bFGF (PeproTech). The medium was changed daily, and the cells were passaged every 5 days using 0.5 mM EDTA (Invitrogen). Human naïve ESCs (nESCs) were cultured in 5iLAF medium containing DMEM/F12:Neurobasal (1:1) (Thermo Fisher), 1% N2 supplement (Thermo Fisher), 2% B27 supplement (Thermo Fisher), 0.5% KnockOut SR (Thermo Fisher), 1% nonessential amino acids (Millipore), 2 mM GlutaMAX (Millipore), penicillin-streptomycin (Millipore), 20 ng/ml human LIF (Millipore), 8 ng/ml bFGF (PeproTech), 50 µg/ml BSA (Sigma) and the following cytokines and small molecules: 1 µM PD0325901 (Selleck), 0.5 µM SB590885 (Selleck), 1 µM WH-4-023 (Selleck), 10 µM Y-27,632 (Selleck), 20 ng/ml activin A (PeproTech) and recombinant human LIF (PeproTech) and were passaged with Accutase (Sigma) every 4–5 days, with daily medium change (Theunissen et al. 2014).

### The primed-to-naïve transition

pESC line carrying the dual reporter system composed of *ALPG*-promoter-RFP (RFP) and *OCT4*-△PE-GFP (GFP) was generated as previously described (Bi et al. 2020; Theunissen et al. 2014). In brief, CMV promoter of pSicoR-RFP plasmid (Addgene) was replaced by the ALPG promoter and plasmids were transiently cotransfected into 293T cells with packing plasmids. After 48 h, the viral supernatants were harvested, concentrated and used for incubating with *OCT4*-△PE-GFP pESCs. For inducing the primed to naïve state transition, 0.5 ~ 1 × 10^5^ dissociated single pESCs were seeded on an irradiated feeder layer in conventional ESC medium supplied with 10 µM Y-27,632 (Selleck). The medium was then switched to 5iLAF medium on the second day and was daily changed. The intermediate cells were collected at different time points during the primed-to-naïve transition using flow cytometry .

### ATAC-seq library generation and sequencing

The ATAC-seq libraries were generated as previously described (Wu et al. 2016). In brief, 5 × 10^4^ cells were washed once with 500 µl cold PBS, centrifuged at 500 g, 4 °C for 5 min, then resuspended in 50 µl lysis buffer (10 mM Tris-HCl (pH 7.4), 10 mM NaCl, 3 mM MgCl_2_, and 0.1% (v/v) NP40 and incubated on ice for 10 min. The suspension with nuclei was then centrifuged for 5 min at 500 g, 4 °C, and 50 µl transposition reaction mixture (10 µl of 5× TTBL, 5 µl of TTE Mix V50 and 35 µl of nuclease-free H_2_O) was added, the mixture was incubated at 37 °C for 30 min. The DNA fragments were isolated by the QIAGEN MinElute kit. ATAC-seq libraries were purified using a QIAquick PCR (QIAGEN) column after 13 cycles of amplification The library concentration was measured using Qubit kit according to the manufacturer’s instructions. Finally, the ATAC library was sequenced on Illumina Novaseq 6000 at Berry Genomics Corporation.

### ChIP-seq library generation and sequencing

For NChIP-seq, 1 × 10^4^ cells were used per reaction, and three replicates were performed for each cell population. All isolated cells were washed three times in 0.5% BSA in PBS (Sigma) to prevent potential contamination. The detailed experimental procedure was performed as previously described The ChIP-seq libraries were generated using the KAPA Hyper Prep Kit for (kk8504) according to the manufacturer’s instructions. 150 bp Paired-end sequencing libraries were generated on Illumina Novaseq 6000 at Berry Genomics Corporation.

### RNA-seq library generation and sequencing

Total RNAs were isolated from cells using TRizol (Invitrogen). The RNA sequencing libraries were generated using KAPA Stranded mRNA-Seq Kit (KAPA) according to the manufacturer’s instructions. 150 bp Paired-end sequencing libraries were generated on Illumina Novaseq 6000 at Berry Genomics Corporation.

### ATAC-seq, ChIP-seq and RNA-seq data processing

The raw ATAC-seq, ChIP-seq and RNA-seq reads were preprocessed to remove adapters and low-quality reads by Trim_galore (version 0.6.6) with default parameters (Martin 2011). The cleaned ATAC-seq reads were aligned to the human genome assembly (hg38) using bowtie2 (version 2.4.1) with default parameters except: “-X 2000 --no-unal --very-sensitive” (Langmead and Salzberg 2012). The cleaned ChIP-seq reads were also aligned to hg38 by bowtie2 with default parameters except: “--no-unal --very-sensitive”. The reads mapped to mitochondrial DNA in BAM files were discarded using the “grep –v chrM” command. For downstream analysis, we only retained the high-quality and concordantly mapped reads using SAMtools view with following options: “–q 30 -f 2” (Li et al. 2009) ,and duplicated reads were removed from the BAM files by sambamba markdup function (version 0.7.1) with “-r” option (Tarasov et al. 2015). The final filtered ATAC-seq and ChIP-seq BAM files were transformed into read coverage files (bigWig format) for visualizationby deepTools (version 3.5.0) with the RPKM normalizationand “--blackListFileName” parameters to remove the hg38 blacklist regions (Ramírez et al. 2016). ATAC-seq peaks were called by MACS2 (version 2.2.7.1) with default options except: “--nomode -f BAMPE --keep-dup all” (Feng et al. 2012). Only peaks detected in at least 2 replicates for each sample were considered and subsequently merged into a single union ATAC-seq peak set by the BEDTools intersect and merge functions (Quinlan and Hall 2010). Motif analyses for each module were performed by the HOMER (v.4.11.1) “findMotifsGenome.pl” function with the “-size given” parameter (Heinz et al. 2010). Peak annotation for the union ATAC-seq peak set was performed by chIPseeker (Yu et al. 2015), which implements functions to retrieve the nearest genes surrounding the peak. For the definition of the “open” or “closed” state of the union ATAC-seq peaks, we first randomly collected the background regions with the same quantity and peak length as the union ATAC-seq peaks from the genome by the BEDTools shuffle function with “-excl” parameter, which excluded the union ATAC-seq peak set regions during regions collection. Then, the ATAC-seq reads of each sample were calculated over the background regions and the union ATAC-seq peaks by the deepTools multiBigwigSummary function with RPKM normalized bigwig files, respectively. The false discovery rate (FDR) were calculated between the peak RPKM matrix and the background RPKM matrix, and we achieved a 1% false discovery rate by setting the peak threshold RPKM value to 25.16. The peaks with a RPKM values below this threshold were annotated to“closed”, while those with its RPKM values above this threshold were annotated “opened”. Gene Ontology analysis was performed using GREAT (McLean et al. 2010).

For the RNA-seq analysis, cleaned reads were mapped to hg38 using STAR with default parameters except:“--outSAMattrIHstart 0, --outSAMstrandField intronMotif, --outFilterIntronMotifs RemoveNoncanonical, --outFilterMismatchNmax 999, --outFilterMismatchNoverReadLmax 0.04, --quantMode GeneCounts, --twopassMode Basic” (Dobin et al. 2013). The FPKM-normalized genes expression level were computed by Stringtie (version 2.1.4) (Pertea et al. 2015). The differential analysis on gene expression was done by DEseq2 using the rawcounts (Love et al. 2014).

### Supplementary Information

**Additional file 1: Figure S1.** Statistics for each module within CAD charting during the primed-to-naive transition. **Figure S2.** Statistics of the differential gene expression for the discordant genes. **Figure S3.** Pileups of the ATAC signals, H3K4me3 signals, H3K27ac signals and RNA signals. **Figure S4.** Signals for the loci of representative genes.

**Additional file 2:** **Table S1.** ATAC-seqPeak annotation, peak RPKM for each module in CAD during the primed-to-naive transition. 

**Additional file 3:** **Table S2.** Mapping rate, quality control for reads and peak numbers of the histone modifications data.

## Data Availability

The bulk RNA-seq datasets and ATAC-seq datasets are available at GEO: GSE174771. The ChIP-seq datasets generated in this study are available at GEO: GSE246440. All data supporting the findings of this study are available within the article and its supplementary information files or from the corresponding author upon reasonable request.

## Abbreviations

ATAC: Assay for transposase-accessible chromatin
TF: Transcription factor
PSCs: Pluripotent stem cells
TE: Trophectoderm
PrE: Primitive endoderm
DNase-seq: DNase I hypersensitive sites sequencing
FAIRE-seq: Formaldehyde-Assisted Isolation of Regulatory Elements sequencing
EGA: Embryonic genomic activation
BiPNT: BMP4 induced primed-to-naïve transition
TFiPNT: TFs induced primed-to-naïve transition
nESC: Naive embryonic stem cell
CAD: Chromatin accessibility dynamics
FDR: False discovery rate
GO: Gene ontology
pESC: Primed embryonic stem cell
ULI-NChIP: Ultra-low-input micrococcal nuclease-based native chromatin immunoprecipitation
