# Supplementary material for: Discordance between chromatin accessibility and transcriptional activity during the human primed-to-naïve pluripotency transition process
Source: Cell Regen. 2023 Nov 8;12:35. doi: 10.1186/s13619-023-00179-2 (PMC10632355; doi:10.1186/s13619-023-00179-2)
Supplement: Supplementary file 1 — Additional file 1: Figure S1. Statistics for each module within CAD charting during the primed-to-naive transition. Figure S2. Statistics of the differential gene expression for the discordant genes. Figure S3. Pileups of the ATAC signals, H3K4me3 signals, H3K27ac signals and RNA signals. Figure S4. Signals for the loci of representative genes. [file 13619_2023_179_MOESM1_ESM.pdf]

Supplemental Information

Figure S1, related to Figure 1.

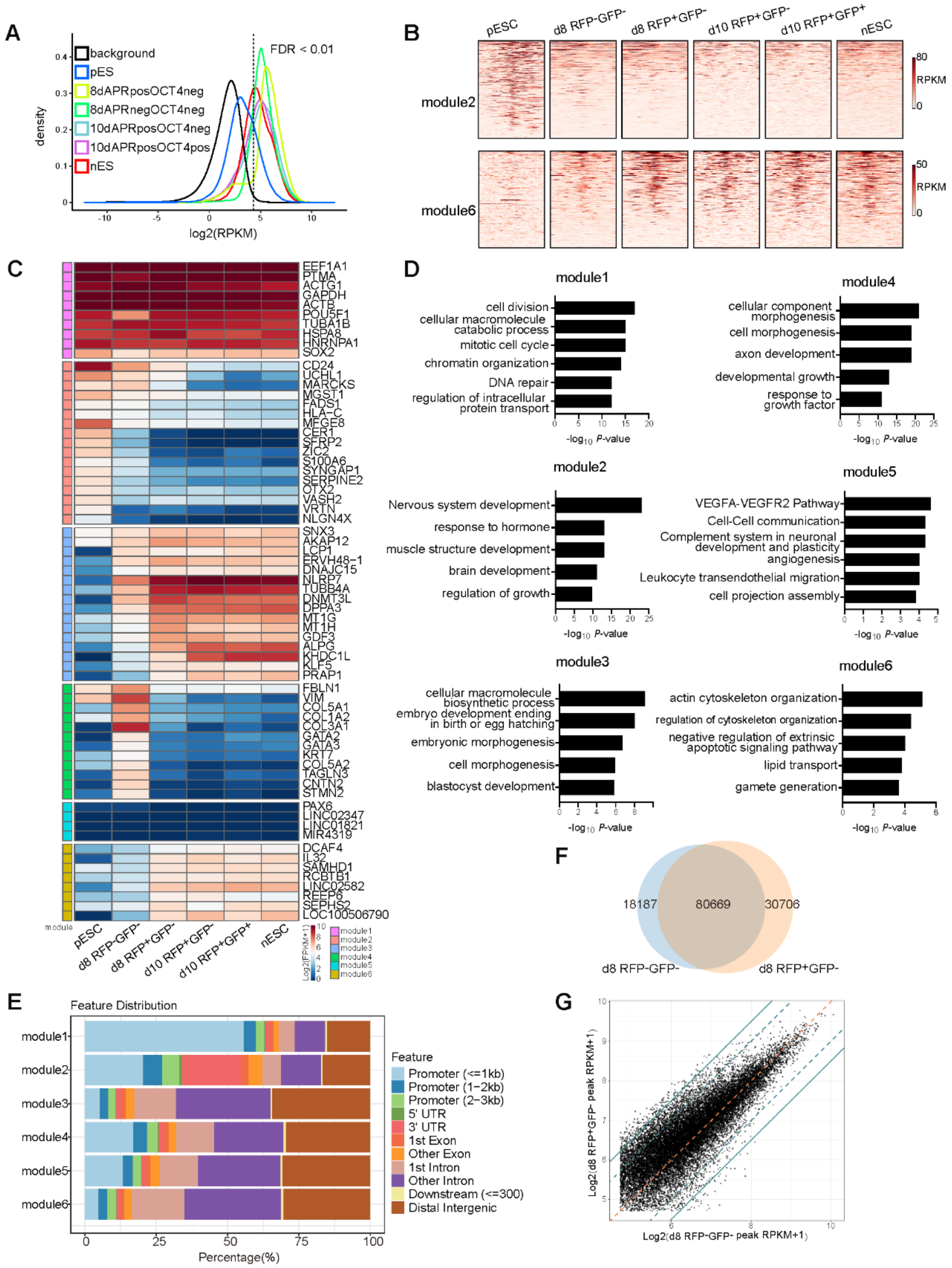

Figure S1, related to Figure 1.

Statistics for each module within CAD charting during the primed-to-naive transition.

(A) Determination of the ATAC-seq signals threshold for defining the opened or closed chromatin. The dashed line represents the cut-off value for FDR < 0.01.

(B) Heatmaps of ATAC-seq signals for module 2 and 6, centered on the ATAC-seq peak (upstream 3 kb and downstream 3 kb of the peaks).

(C) Heatmap showing the representative genes expression of each module during the primed-to-naive transition process.

(D) Bar plots showing enrichment of *p*-value for representative gene ontology (GO) terms of each module.

(E) Peak annotation and distribution for each module.

(F) Venn diagram for the overlap of peak between RFP-negative and RFP-positive cells.

(G) Scatterplot showing the signal strength (RPKM) comparison of peaks opened in both RFP-positive and RFP-negative on day 8. Blue dashed lines: $| \log2 (\text{RFP-positive} / \text{RFP-negative}) | < 1$; Blue solid lines: $| \log2 (\text{RFP-positive} / \text{RFP-negative}) | < 1.5$; Orange dashed line denotes equal RPKM.

Figure S2, related to Figure 2.

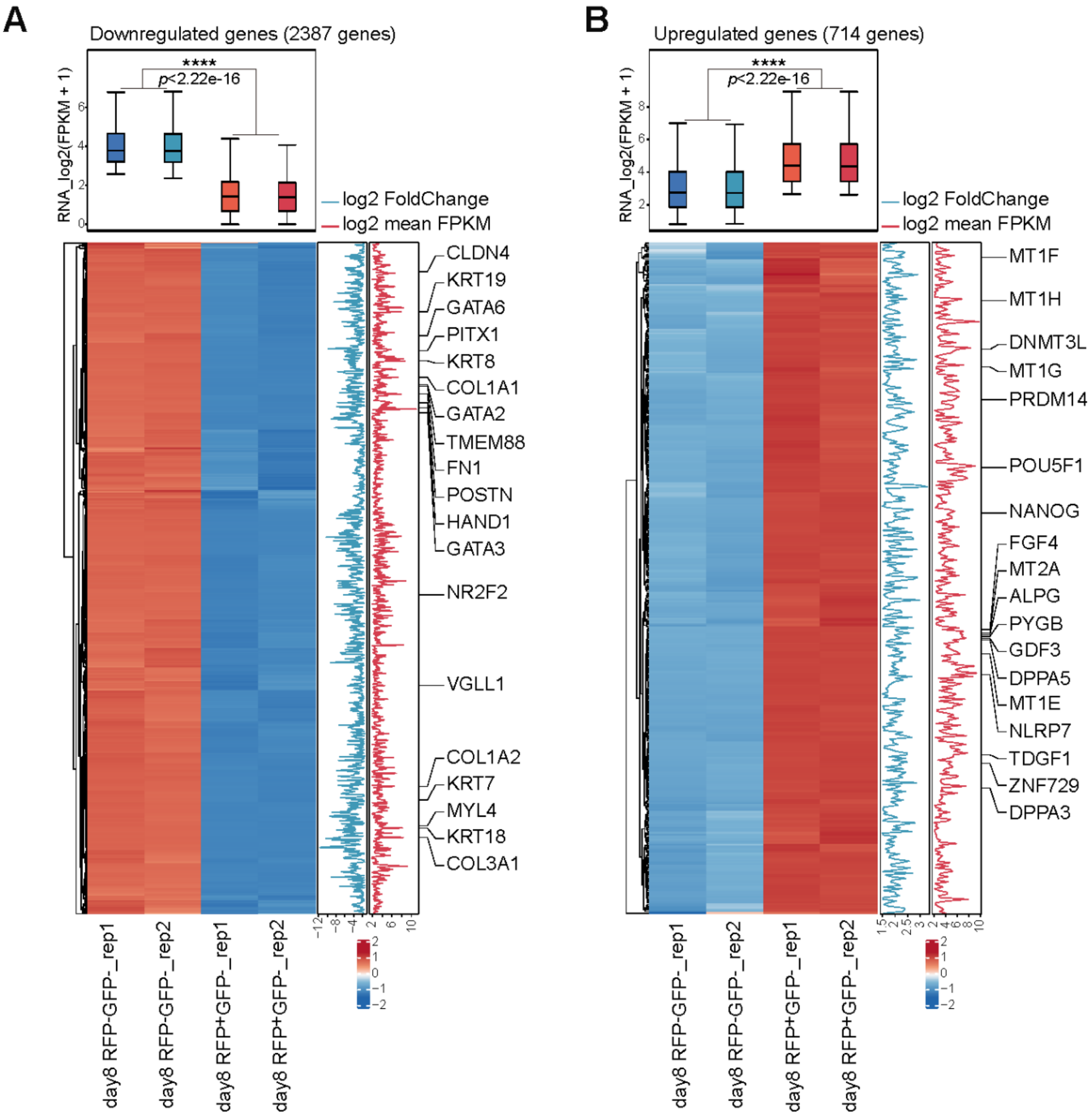

Figure S2, related to Figure 2.

Statistics of the differential gene expression for the discordant genes.

(A) Heatmap showing the foldchange and mean FPKM across samples for the discordant genes with a higher expression level in RFP-negative cells, with representative genes listed in the right panels and comparison for mean FPKM of each sample listed in the upper panel.

(B) Heatmap showing the foldchange and mean FPKM across samples for the discordant genes with a higher expression level in RFP-positive cells, with representative genes listed in the right panels and comparison for mean FPKM of each sample listed in the upper panel.

Figure S3, related to Figure 3.

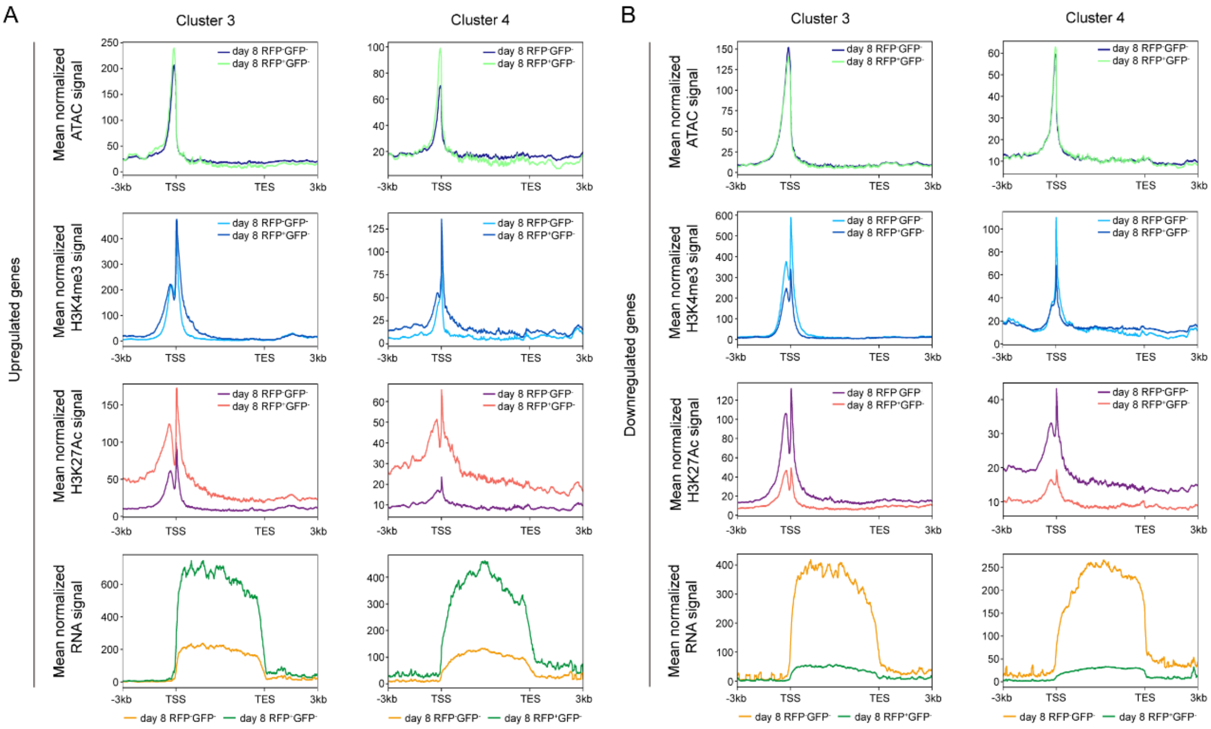

Figure S3, related to Figure 3.

Pileups of the ATAC signals, H3K4me3 signals, H3K27ac signals and RNA signals.

(A) Pileups of mean ATAC signals (the first panel), mean H3K4me3 signals (the second panel), mean H3K27ac signals (the third panel) and mean RNA signals (the fourth panel) for the genes within cluster 3 and cluster 4 as presented in Figure 3A (upstream 3 kb and downstream 3 kb of the peaks or transcripts with merged exons).

(B) Pileups of mean ATAC signals (first panel), mean H3K4me3 signals (second panel), mean H3K27ac signals (third panel) and mean RNA signals (fourth panel) for the genes within cluster 3 and cluster 4 as shown in Figure 3C (upstream 3 kb and downstream 3 kb of the peaks or transcripts with merged exons).

Figure S4, related to Figure 3.

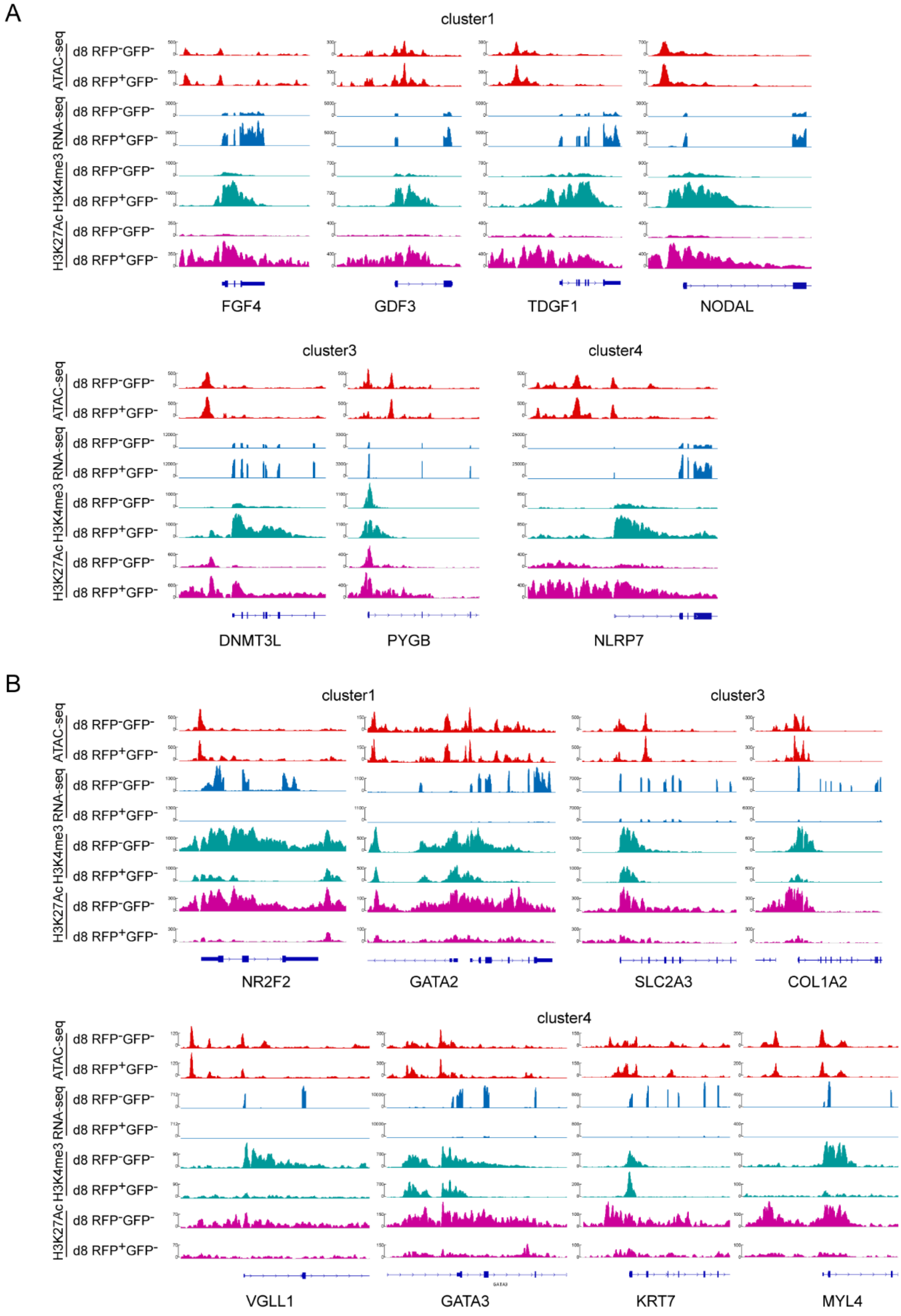

Figure S4, related to Figure 3.

Signals for the loci of representative genes.

(A) ATAC signals (the first panel), RNA signals (the second panel), H3K4me3 signals (the third panel) and H3K27ac signals (the fourth panel) for the representative loci within cluster 1, cluster 3 and cluster 4 as shown in Figure 3A.

(B) ATAC signals (first panel), RNA signals (second panel), H3K4me3 signals (third panel) and H3K27ac signals (fourth panel) for the representative loci within cluster 1, cluster 3 and cluster 4 as shown in Figure 3C.
